# New evidence for supplementary crop production, foddering and fuel use by Bronze Age transhumant pastoralists in the Tianshan Mountains

**DOI:** 10.1038/s41598-021-93090-2

**Published:** 2021-07-02

**Authors:** Duo Tian, Marcella Festa, Dexin Cong, Zhijun Zhao, Peter Weiming Jia, Alison Betts

**Affiliations:** 1 Institute of Middle Eastern Studies, Northwest University, Xi’an, 710127 Shaanxi People’s Republic of China; 2 School of Cultural Heritage, Northwest University, Xi’an, 710127 Shaanxi People’s Republic of China; 3 Institute of Archaeology, Chinese Academy of Social Sciences, Beijing, 100010 People’s Republic of China; 4 Department of Archaeology and China Studies Centre, University of Sydney, Sydney, NSW 2006 Australia; 5 School of History and Culture, Henan University, Kaifeng, 475001 People’s Republic of China

**Keywords:** Anthropology, Archaeology

## Abstract

The nature of economies and the movement of agricultural crops across Eurasia in the Bronze Age have been the subject of significant research interest in recent years. This study presents and discusses new results of flotation, radiocarbon and carbon stable isotope analyses from the seed assemblage at the Adunqiaolu site (northwestern Xinjiang), in combination with archaeological evidence. Archaeobotanical evidence, including carbonized foxtail millet, broomcorn millet, and naked barley, documents the diversity of local cereal consumption during the mid-second millennium BC. Our results suggest that crops were not grown locally, however, but in the lower Boertala Valley, supporting the argument that Adunqiaolu was a winter camp. These new sets of data constitute an important contribution to the discussion on cereal dispersal across the Tianshan Mountains in the Bronze Age.

## Introduction

The Bronze Age of Eurasia has been associated with increasing interaction between different populations^1–3^. A growing body of research focusing on ‘food globalization’ in Eurasia^4–7^ has pointed to Central Asia, and in particular to the Inner Asia Mountain Corridor (henceforth IAMC) as a significant route for the exchange and diffusion of cereals^8–17^. The IAMC is a foothill ecozone ranging from the Hindu Kush, through the Pamir, Tianshan, and Dzhungar Mountains, to the Altai Range, which functioned as a pathway for the transmission of cultural and economic practices^9^.

**Figure 1:**
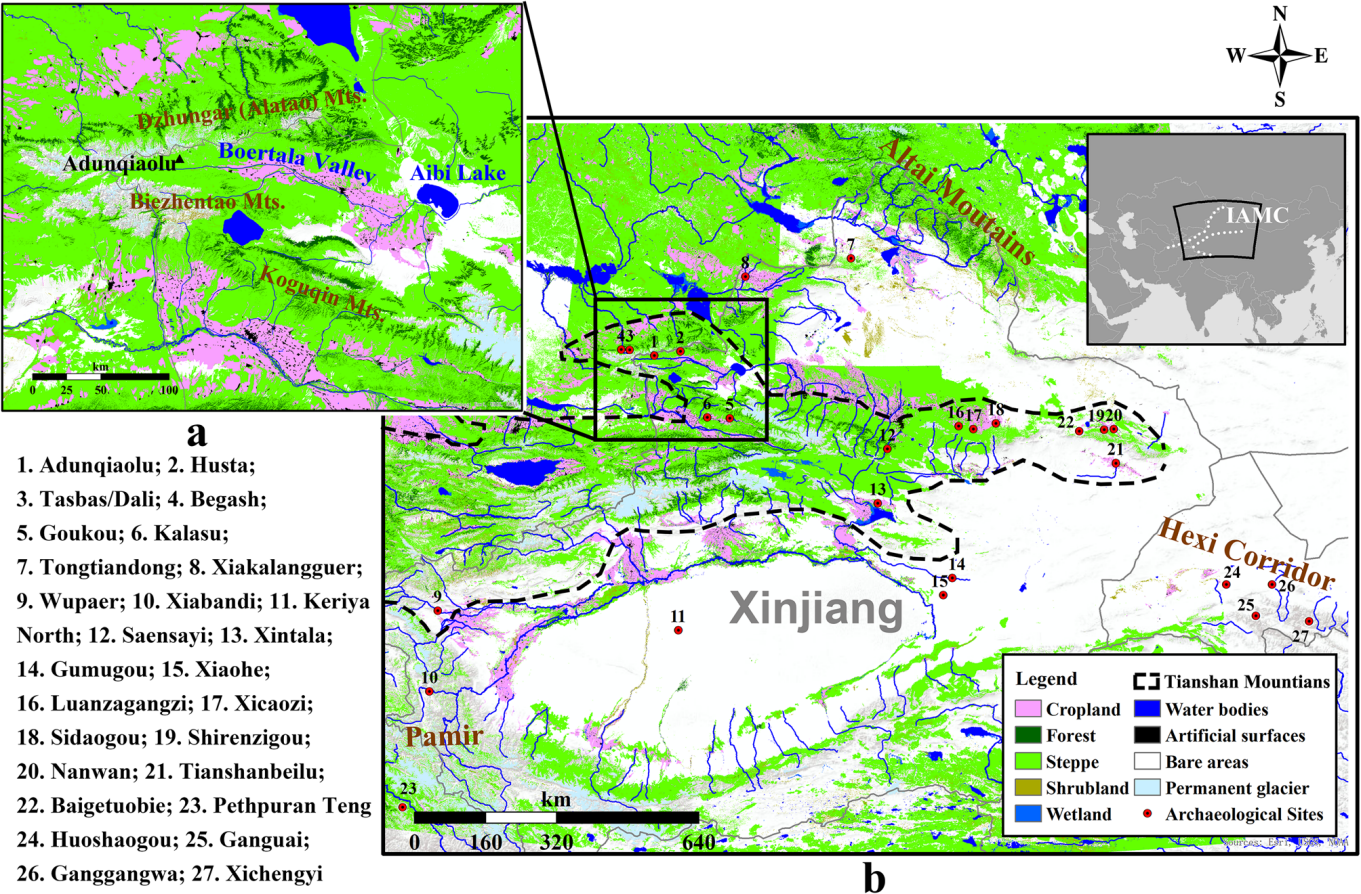
Map of the sites mentioned in this study and the modern landcover of Xinjiang (this map is edited by D.T. using ArcGIS 10.6 https://www.esri.com, the base map is from World Terrain Base in ArcGIS Online https://www.arcgis.com/home/item.html?id=d476e726bd8c4c3aa5168d735f647dcc, and the map of modern landcover is adapted from open access map GlobeLand30 http://www.globeland30.org/home_en.html, introduced by Chen et al^87^).

Early evidence of crops consists of a few grains of wheat and barley from the Altai region (i.e., Tongtiandong Cave, Jeminay County) dated to the late fourth millennium BC^18^. However, a consistent expansion and diversification of crop use occurred in the IAMC in the period 2500–1500 BC^7,19,20^. A variety of grains—including naked barley, broomcorn millet, wheat and pea—were recovered from archaeobotanical assemblages in present-day Kazakhstan, showing dietary diversity in the regional Bronze Age communities^21,22^. In the Hexi Corridor, at the easternmost end of the Tianshan Range, the traditional C4 millet-based diet was integrated with barley and wheat after 2000 BC, as demonstrated by isotopic and macro-botanical data-sets^23,24^ (Figs. 1, 6). While researchers generally agree that cereals spread across Central Asia from their original sites of domestication in East and West Asia, there is an ongoing debate as to how and why this bidirectional crop dispersal occurred, probably as early as the mid-3rd millennium BC^7,19,25–27^. This study presents new results of the flotation work conducted at Adunqiaolu (Wenquan County, northwest Xinjiang), combined with new radiocarbon dates and carbon stable isotopes. The novel datasets are discussed in light of the archaeological material in order to contribute to a better understanding of crop dispersal via the IAMC and, specifically, the Tianshan Mountains during the Bronze Age.

The Bronze Age settlement of Adunqiaolu is located in the southern foothills of the Alatao (Dzunghar) Mountain, on the upper reaches of the Boertala River Valley (Fig. 1). According to the altitude, the area is characterized by three vegetation zones: at about 1800 masl the narrow and rocky river valley is surrounded by a few wooded areas (e.g., *Salix* spp., *Ulmus* spp.) and clusters of shrubs (e.g. *Caragana sinica*, *Achnatherum splendens*, *Hippophae rhamnoides*); at ca. 2300 masl steppe plants (e.g. *Stipa capillata*, *Convolvulus fruticosus*, *Festuca rupicola* and various Cyperaceae, Asteraceae, Fabaceae and Liliaceae) dominates the environment^28^. Above 3000 masl, the area is characterized by alpine deserts and glaciers.

**Figure 2:**
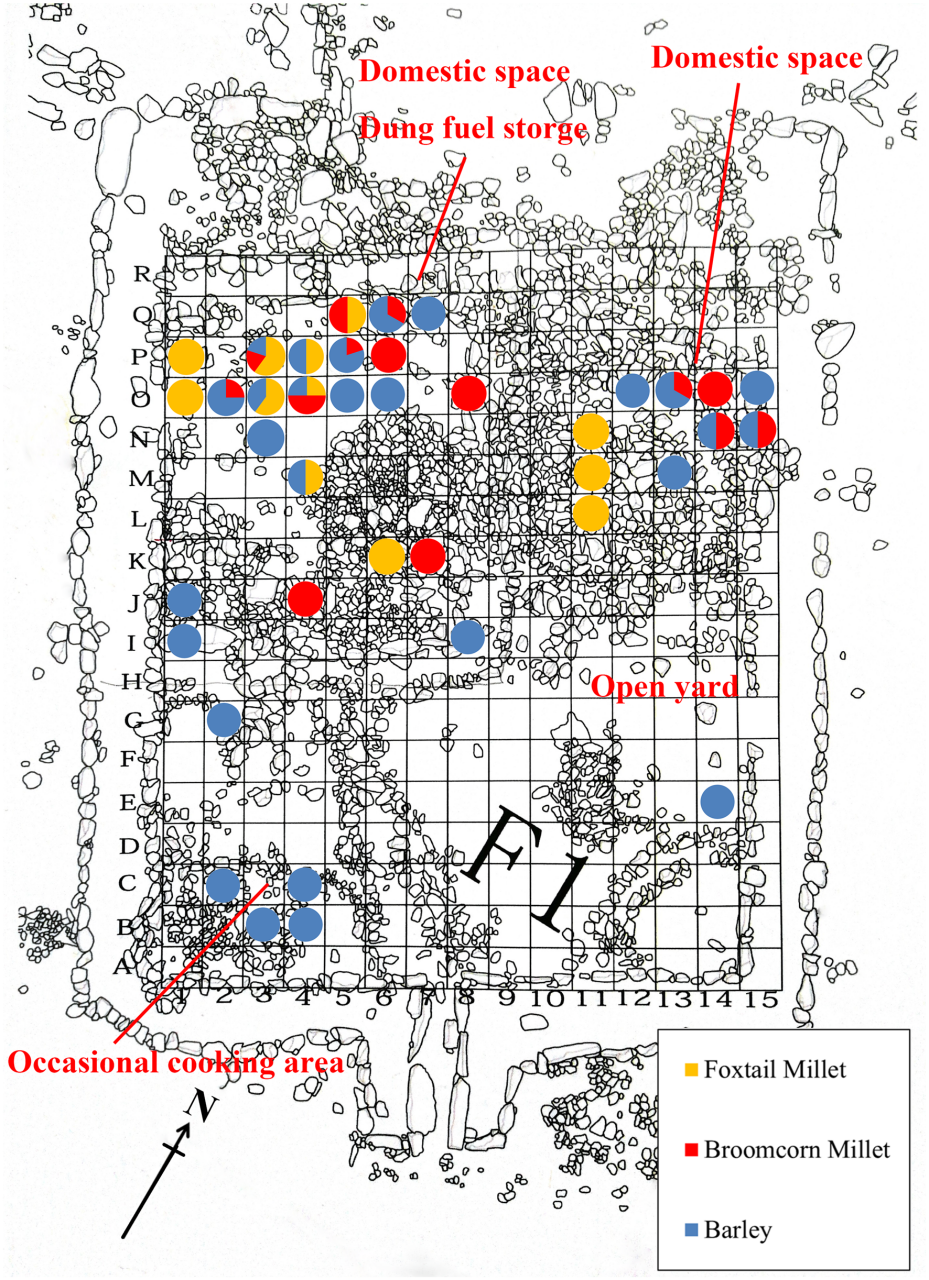
Plan of F1 and quantitative proportions of cereal crops per sampling grids (the plan is mapped by D.C. in-field and digitized using Adobe Photoshop CC 14.0 https://www.adobe.com/products/photoshop.html).

During the survey and excavations at Adunqiaolu in 2011–2014, eleven groups of houses were discovered. Based on analogy with present-day pastoral land use and analysis of pollen and phytoliths from ancient sheep dung, at least one house, F1, at an altitude of 2300 masl, was most likely a winter settlement, occupied only on a seasonal basis^29,30^. The botanical evidence examined in this paper was recovered from F1, which was a rectangular semi-subterranean structure, framed by a double line of stone slabs and internally divided into four spaces (Fig. 2). Ash pits, fireplaces, pottery sherds, stone artifacts and dung were found. There was evidence for a differential use of space within the structure. The north-west section appears to have been a domestic space with a large hearth, a scatter of potsherds and well-preserved animal dung, possibly stored for fuel. The north-east section too perhaps was predominantly intended for domestic use, as it contained sherd scatters but no hearth. The south-east section may have been an open yard. It had no artefacts but contained a large flat rock at the base. The south-west corner contained a dense concentration of sherds in a matrix of ash with traces of burning possibly from occasional cooking. House F1 yielded a sequence of radiocarbon dates showing that it was in use for around 400 years from the mid-eighteenth century BC onward^31,32^ (Table 1). The radiocarbon dates, artifacts and architecture at Adunqiaolu are all consistent with the eastern Andronovo archaeological complex (Federovo variant)^31^ (Table 1).

**Table 1:** AMS C^14^dates and Δ^13^C value for F1, Adunqiaolu. Dating of barley is published in this article for the first time; dates of charcoal and dung have been reported from Jia et al.^31^.

Lab No	Context	Material	Conventional Radiocarbon date (BP)	Calibrated Dates at 95.4% (BC)	δ^13^C (‰)	Δ^13^C
Beta-367941	Layer 3 (Burnt floor–N3)	Barley	3060 ± 30	1389–1282	− 21.0	14.8
Beta-520039	Layer 4 (Fireplace–O2)	Barley	3260 ± 30	1616–1454	− 23.3	17.3
Beta-520040	Layer 4 (Fireplace–N14&N15)	Barley	3200 ± 30	1526–1417	− 24.2	18.2
UBA-19163	Layer 2	Charcoal	3331 ± 38	1642–1611		
UBA-19164	Layer 3	Charcoal	3270 ± 27	1607–1506		
UBA-19165	Layer 4	Charcoal	3403 ± 28	1743–1666		
UBA-30786	P5-1	Dung	3251 ± 33	1531–1501		
UBA-30781	P5-2	Dung	3189 ± 37	1496–1433		
UBA-30783	P7	Dung	3090 ± 28	1410–1304		
UBA-30789	O8	Charcoal	3265 ± 32	1601–1503		

## Results

### Seeds

1174 carbonized seeds were collected from 85 of the 110 samples, with a density of 0.79 per liter of soil sample. 71 domesticated grains were recovered from 39 samples, accounting for 6.0% of the total. They include naked barley, broomcorn millet and foxtail millet (Fig. 2). The assemblage is consistent with that of the phytoliths examined by Shao et al.^33^. 1103 seeds of herbaceous plants (94.1%)—10 *genera* from 7 families—were also identified (Table 2 and Table S1).

**Table 2:** Seeds in F1: taxonomy, quantity and ubiquity.

Taxa	Quantity	Proportion		Ubiquity
**Domesticates**				
*Setaria italica*	17	1.4%		10.9%
*Panicum miliaceum*	16	1.4%		12.7%
*Hordeum vulgare* (intact)	19	1.6%		23.4%
*Hordeum vulgare* (fragmented)	19	1.6%		
**Others**				
Poaceae				
* Setaria viridis*	2	0.2%		1.8%
* Avena fatua*	5	0.4%		4.5%
**Fabaceae**				
*Astragalus* sp.	468	39.9%		70.0%
**Chenopodiaceae**				
*Chenopodium album*	601	51.2%		40.9%
*Salsola* sp.	2	0.2%		1.8%
*Atriplex* sp.	10	0.9%		7.3%
**Cyperaceae**				
*Carex* sp.	5	0.4%		2.7%
**Rubiaceae**				
*Galium* spp.	2	0.2%		1.8%
**Polygonaceae**				
*Rumex* sp.	1	0.1%		0.9%
**Valerianaceae**				
*Valeriana* sp.	1	0.1%		0.9%
Unknown	6	0.5%		4.5%
Total	1174			110 samples

#### Barley (Hordeum vulgare)

**Figure 3:**
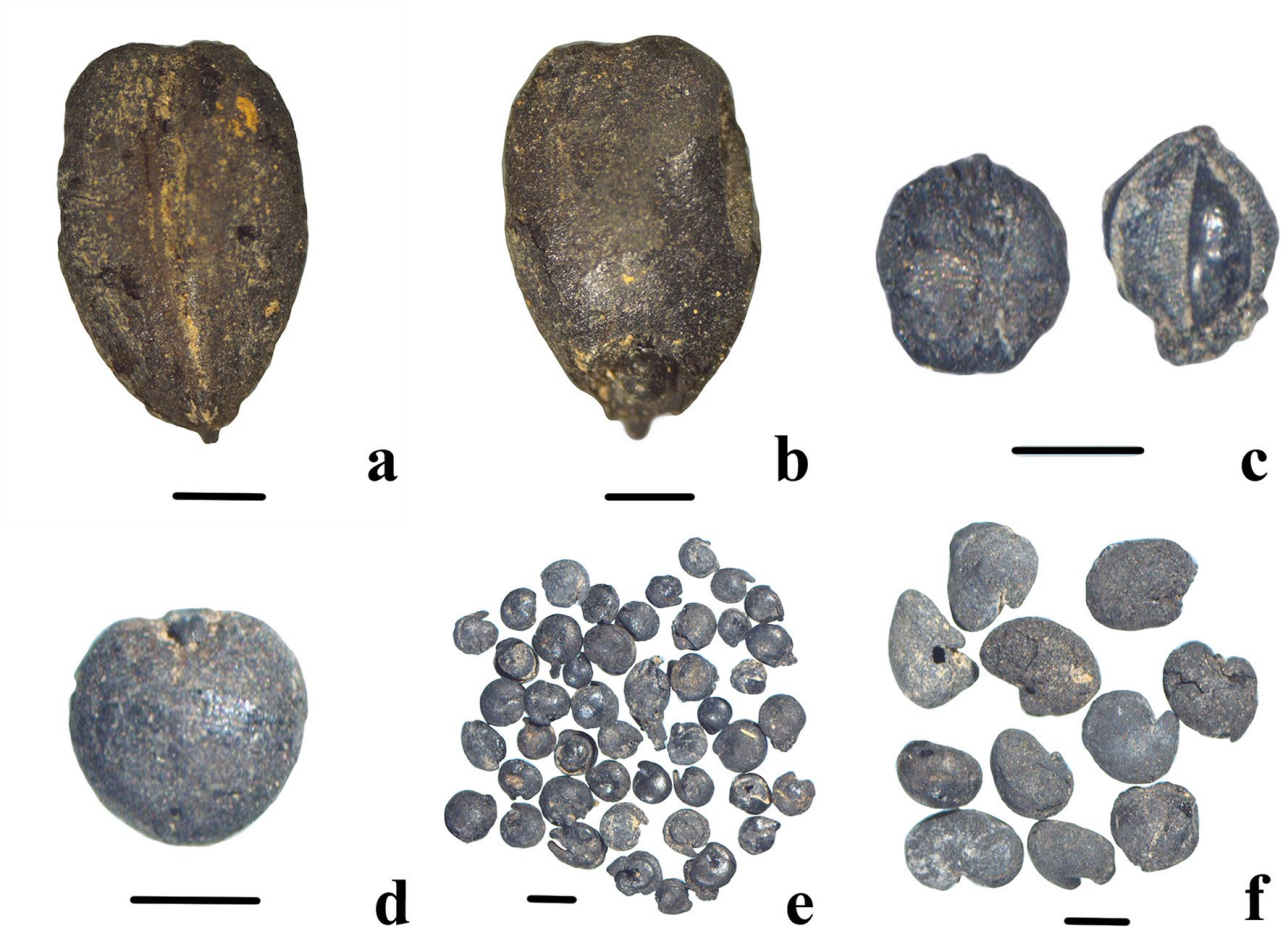
Seeds from F1. Scale bar is 1 mm. (**a**) Barley (ventral side); **(b)** Barley (dorsal side); **(c)** Foxtail millet; **(d)** Broomcorn millet; **(e)** *Chenopodium album*; **(f)** *Astragalus* sp. (the figure is generated by D.T. using Adobe Photoshop CC 14.0 https://www.adobe.com/products/photoshop.html).

Naked barley is the most abundant and ubiquitous cereal in Adunqiaolu (intact grains = 19; fragments = 19) (Fig. 3a,b). The average length and width of the grains are 4.26 mm and 2.81 mm, respectively (Table S3). The twisted shape indicates that it is six-row barley^34^. Notably, no by-products of crop processing, such as husks and rachises, were found.

#### Millets

Foxtail millet (*Setaria italica*) (n = 17) (Fig. 3c) and broomcorn millet (*Panicum miliaceum*) (n = 16) (Fig. 3d) reach similar levels of ubiquity. The average length and width of broomcorn millet grains are 1.94 mm and 1.78 mm respectively, and those of foxtail millet grains are 1.48 mm and 1.31 mm respectively (Table S3 and Table S5). The florets of foxtail millet—a caryopsis with a husk—are exceptionally well-preserved and match the criteria for identification ^35^.

#### Other seeds

With the exception of a few plant seeds of Polygonaceae, Rubiaceae and Valerianaceae, the other herbaceous seeds are forage *genera* existing in Xinjiang^36,37^. Among them, *Chenopodium album* (Fig. 3e) and *Astragalus* sp. (Fig. 3f) present a high degree of ubiquity.

### Chronology

The results of radiocarbon dating are consistent with those obtained by Jia et. al^31^, located in the mid-second millennium BC (Table 1).

### Carbon stable isotopes

The δ^13^C values of three barley samples range from − 21.0‰ to − 24.2‰, while the calculated Δ^13^C values range from 14.9‰ to 18.2‰ (Table 1).

## Discussion

### Subsistence strategies in Adunqiaolu

The archaeobotanical evidence indicates a multi-crop consumption pattern in Adunqiaolu in the mid-second millennium BC. Accelerator Mass Spectrometry (henceforth AMS) dating for barley grains ranges from the late seventeenth century BC (Layer 4) to the thirteenth century BC (Layer 3) (Table 1). The broomcorn millet and foxtail millet remains have not been dated by AMS; they co-exist with barley in the samples collected from the floor near the fireplaces (grids O2, O3, O4 and P3, Fig. 2 and Table S1).

Botanical material indicates that the occupants of Adunqiaolu consumed agricultural products. However, there is little evidence for the local cultivation of such products. The rocky high mountain steppe landscape of Adunqiaolu is poorly suited to crop cultivation. The ancient site is located at 2300 masl, while evidence of Bronze Age agricultural practices in the IAMC have been mostly found below 2000 masl^38,39^. Most importantly, Adunquiolu has been identified as a winter camp^29,30^ where cultivation would not be expected. The area of pasture where it is located is regarded as an optimal site for winter as it is in a sheltered position on a south-facing slope and has a particular localised wind pattern that prevents snow from drifting deeply. This allows animals to maximize their access to grazing in the cooler months and thus reduces the amount of fodder needed to support them. The low density of cereal grains and the absence of by-products of crop processing, crop residues and farming tools in the archaeological context are consistent with the argument that Adunqiaolu was exclusively a pastoral site^40–42^. House F1 lies immediately adjacent to a modern seasonal winter camp comprising a stone-built house with animal pen. Within the modern annual cycle employed by Kazakh and Mongol herders, there is a dominant focus on seasonal transhumant pastoralism, but agriculture is practiced for food, cash crops and winter fodder in low-lying camps on level ground, close to the river, that is occupied in spring and late summer-autumn^30^. It is likely, therefore, that crops from F1 were grown on lower altitude sites, possibly on the fertile lacustrine plain, in the lower reaches of the Boertala Valley, where the river flows into Lake Aibi and the valley flattens at an elevation of 400–600 m (Fig. 1a). This area, ca. 80 km east of ancient Adunqiaolu, is characterized by fertile soils and relatively abundant water. It is an important area for modern agriculture^43^ and recent archaeological surveys have identified Bronze Age sites^40^.

**Figure 4:**
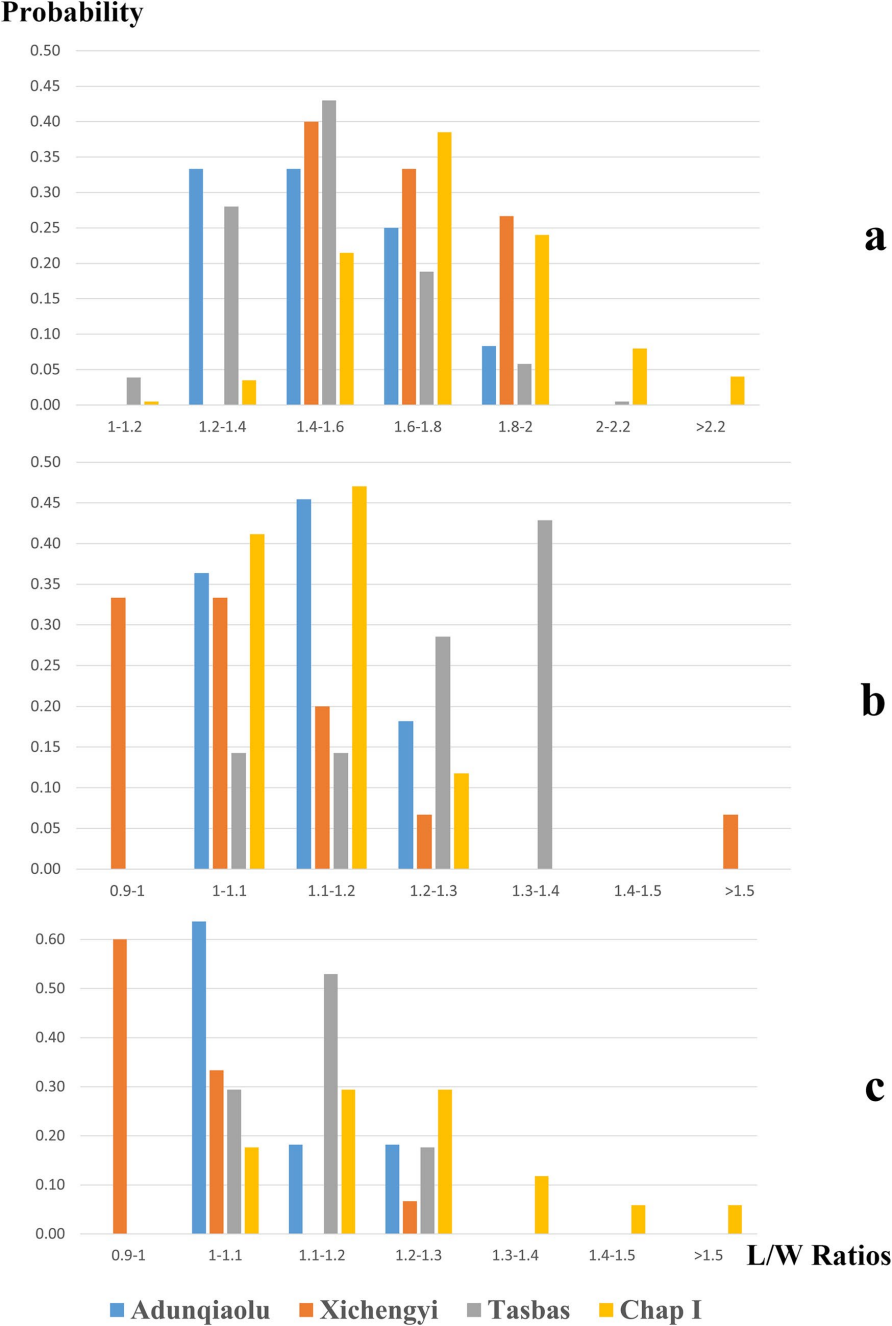
The distribution of Length to Width (L/W) ratios of cereal crops in Bronze Age sites in Xinjiang, Eastern Kazakhstan and Hexi Corridor. (**a**) Barley; **(b)** Foxtail millet; **(c)** Broomcorn millet (the figure is generated by D.T. using Microsoft 365 Excel 2104 https://www.microsoft.com/en-us/microsoft-365/enterprise).

The low standard deviation (SDEV) of grain size calculated for crops recovered from F1 shows relatively small variability (Table S3, Table S4 and Table S5). The size range is consistent with measurements recorded in Bronze Age agricultural sites at low elevation (below 1500 masl). L/W ratio values for barley grains in Adunqiaolu are close to those recorded in Tasbas; values for millet are comparable to those of grains from the Hexi Corridor (Fig. 4)^44,45^. Agricultural sites at higher altitude (2000 masl), such as Chap I, show more variety in grain size (Fig. 4)^46^.

Our carbon stable isotope results are limited by the low number of samples (n = 3); however, they still provide a rough estimate of the quantity of water available to the crops. Δ^13^C values fall in the poor (< 17‰) to moderate (17–18.5‰) water input range according to Wallace et al.^47^. This suggests a limited availability of water in the absence of advanced water-management systems. The comparison of Δ^13^C values with the AMS dates shows a decline in water supply from the mid-second millennium BC. This may possibly be related to the decline in precipitation after 1500 BC^48^. However, further carbon stable isotope investigations are necessary to clarify specific regional water conditions in relation to cereal cultivation.

**Figure 5:**
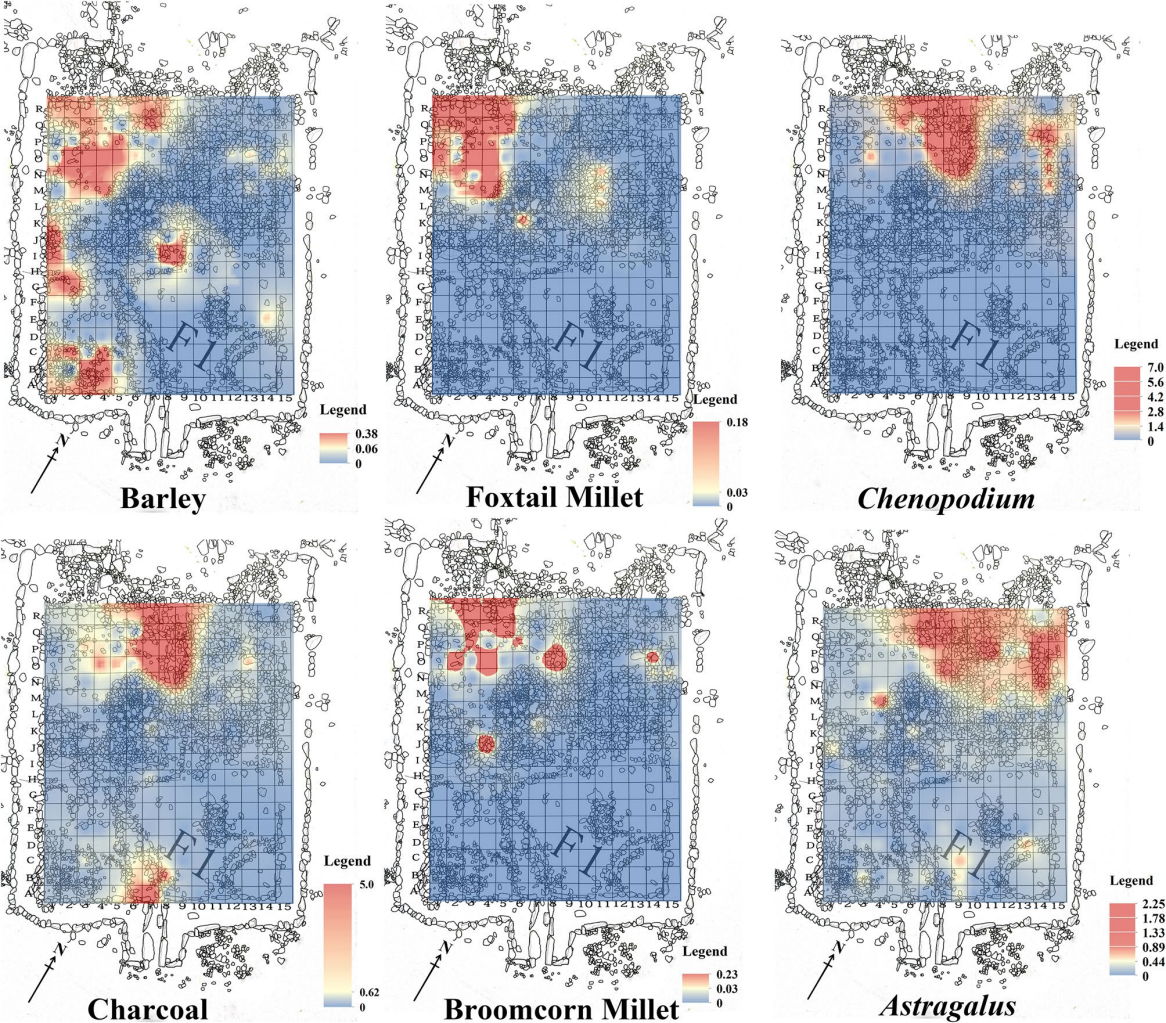
Density of charcoal, *Chenopodium, Astragalus,* barley and millet in F1 (the base map is Fig. 2, and the distribution of density is calculated and illustrated by D.T. using ArcGIS 10.6 https://www.esri.com)*.*

Among the non-domesticated plants, charred remains of *Chenopodium* were discovered in F1. *Chenopodium* is widespread in Central Asia, including Xinjiang, owing to its long fruit-growing season^49^ hard testa and reproductive rate^50^. In particular, it grows densely on abandoned campsites^51^. In the Bronze Age and throughout the Iron Age, *Chenopodium* was similarly distributed^18,52^. Spengler^51^ has argued that high representation of *Chenopodium* in Central Asian sites is a result of the use of dung for fuel. Figure 5 and Table S1 illustrate the high density of *Chenopodium* in the domestic space where dung fossil is found. It can therefore be suggested that some of the herbaceous seeds found in F1 were deposited when animal dung containing them was burned on site.

*Astragalus* is not a tough or high-yield herb. Hence, the considerable amounts of it found in F1 are likely to be at least partially related to the practice of storage and use as fodder for livestock. These seeds of Fabaceae may have found their way into the fire either as debris on the floor or contained within dung used as fuel (Figs. 2, 5). Eleven species, distributed across the river floodplains and mountain meadows in Adunqiaolu, are still used as forage by local herders^36,49^. Ethnographic data from Tuva and the Altai indicate that local pastoralists would collect wild grass or shrubs as supplementary food for herding before moving to their winter camps^53^. Archaeobotanical evidence from the Trans-Urals also suggests that Fabaceae were stored as forage in winter settlements^54^.

Within F1, there is a clear distinction between the distribution of *Chenopodium* and that of *Astragalus* (Fig. 5), implying differential storage patterns and probably use. *Chenopodium* remains closely match those of general charcoal distribution, both occurring in the area of dung storage against the center of the back wall of the building. This perhaps suggests the presence of waste fuel from hearths in the adjacent domestic space in the north-west corner. *Astragulus* is more widely dispersed within the north-east corner, reinforcing the idea that it was collected and stored separately for fodder. In this case, it might seem odd that carbonized seeds were present, but there is evidence that F1 was abandoned following a fire. Burned timbers were found in this area which may have fallen from either the roof or from a burnt wooden internal partition. It might then be suggested that the distinct differences in the distribution of *Chenopodium* and *Astragalus* reflect patterns of storage at the time of the fire.

Despite the relatively small samples, there are also significant differences in the distribution of barley and millet. Both appear to have been stored and used in the northwest corner of the building, but barley was more widely dispersed, including the cooking area to the south-west, with extra evidence for storage in a small chamber in the center of the structure. These patterns may imply that barley was used more for human consumption while millet may perhaps have served as supplementary fodder as well as occasional food. This suggestion is supported by studies of isotopic ratios in faunal remains at the sites of Begash and Dali in the eastern mountains of Kazakhstan not far over the border from Adunqialou^27^.

### Adunqiaolu and crop dispersal

Radiocarbon-dated archaeobotanical evidence suggests that wheat and barley moved northeastward from the Karakorum Mountains to the Altai in the late fourth millennium BC^18^, while the IAMC possibly begun to function^9^. They then spread to Central China via various routes^16,26,55,56^. As a result, these crops were consistently consumed in the second millennium BC in the Tianshan Mountains, Hexi Corridor and Tibetan Plateau^14,23,57^. Macro-botanical data show that barley was more popular than wheat in the mountain areas of Xinjiang, perhaps owing to its ecological versatility, its adaptation to high-altitude environments and the scheduling requirements of mountain pastoralism^52^. In F1 no wheat grains were found.

Broomcorn millet and foxtail millet have a comparatively lower density and lack radiocarbon dates. Convincing Bronze Age evidence of foxtail millet in Xinjiang includes the charred remains in Tongtiandong, dated to the early second millennium BC^18^, and the grains from Adunqiaolu presented in this study. Further west, carbonized foxtail millet remains and phytolith fossils from Tasbas 2a have been placed after 1500 BC^38^, which fits the general date range for foxtail millet consumption in Central Asia. Early carbonized broomcorn millet was recovered from Begash and Dali, ca. 250 km west of Adunqiaolu, where it was most likely used for rituals and forage^27,58^. A few charred grains were found at Tongtiandong^18^. They all date from the late third millennium BC. Broomcorn millet directly dated to c. 2500–2400 BC has recently been reported from Pethpuran Teng, a Northern Neolithic site in Kashmir, suggesting that in addition to the movement of millet from northwest China into Semirech’ye and the Altai, there may have also been an early transmission into the Western Himalayas, southwards along the IAMC^19^. Large quantities of broomcorn millet grains discovered in Xiaohe (Lop Nur) were used for ritual purposes and as staple food in the early second millennium BC^59–61^. Genetic studies and stable carbon isotopic research on ancient crops and diets have provided further evidence for millet diffusion in Xinjiang and Central Asia after 2000 BC^22,62–65^. This is corroborated by the new data from Adunqiaolu presented in this study.

A consistent and relatively large-scale consumption of diverse crop grains is especially attested in the foothills of the Tianshan Mountains in the second millennium BC^39,66^, (Table 3). This steppe/mountain ecozone can support herding and low-investment farming and it is important for agro-pastoralism. During the late Holocene, the climate in the Tianshan Mountains fluctuated slightly between warm and cool phases, and no radical change has been recorded^67,68^. Indeed, the distribution of modern croplands on the hilly flanks of the Tianshan Mountains generally overlaps with that of the Bronze Age sites where crops remains have been found (Fig. 1b). Modern villages on the northern slopes of the Tianshan Mountains are located on river terraces in the foothills or on floodplains where the rainfall is higher and temperatures less extreme than further south. Rainfall along the foothills is around 350 mm, within the practical range for dry agriculture^69^. Notably, however, on local scales the fluctuation of temperature has affected crop productivity. In particular, the diversification in cropping patterns across Central Asia in the second millennium BC has been linked to the need to deal with cooler conditions^70^.

**Table 3:** AMS C^14^dates of cereal crops in Bronze Age sites in Xinjiang and and eastern Kazakhstan. Dates calibrated in OxCal 4.3, using IntCal13 calibration curve^83,84^.

Site	Material	Calibrated Date at 95.4% (BC)	References
Tongtiandong	Wheat	3262–2917	Zhou et al.^18^
	Barley	3347–3097	
	Barley	3336–2945	
	Barley	2461–2210	
	Broomcorn Millet	2199–1981	
	Broomcorn Millet	1623–1460	
	Broomcorn Millet	1623–1460	
Xintala	Wheat	1972–1694	Dodson et al.^10^
Wupaer	Wheat	1506–1303	
	Wheat	1186–909	Yang et al.^20^
	Wheat	1506–1300	
	Barley	1501–1320	
Xicaozi	Wheat	1381–1047	Dodson et al.^10^
Sidaogou	Wheat	1496–1132	
	Barley	975–831	Liu et al.^16^
Xiaohe	Wheat	1896–1697	Liu et al.^14^
	Broomcorn Millet	2011–1756	Flad et al*.*^8^
Gumugou	Wheat	1886–1746	Liu et al.^14^
Keriya North	Wheat	1879–1625	Mair^88^
Adunqiaolu	Barley	1616–1454	This study
Begash	Broomcorn Millet & Wheat	2461–2154	Frachetti et al.^55^
Tasbas	Wheat	2617–2468	Doumani et al.^38^
	Barley	1437–1233	
	Barley	1405–1132	

**Figure 6:**
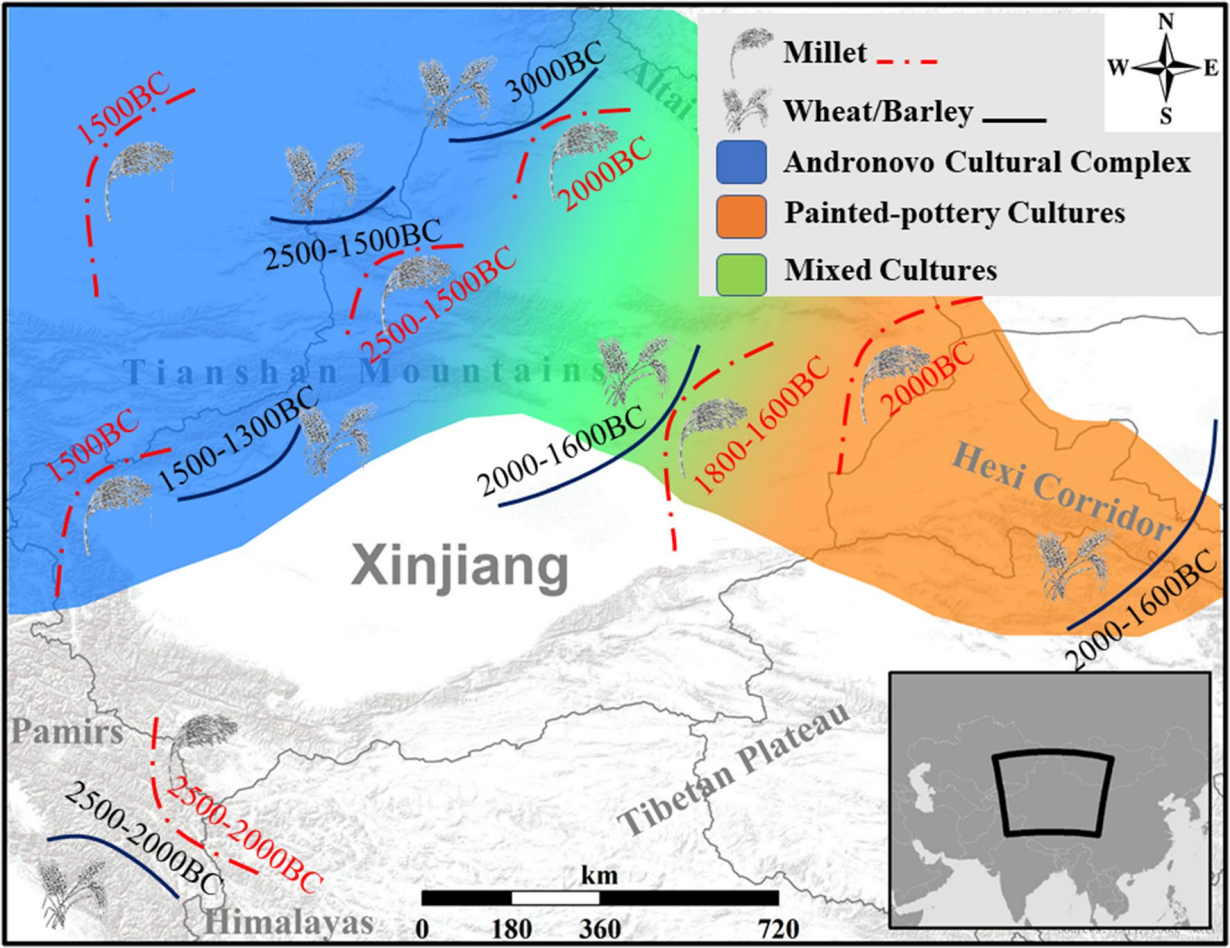
Diffusion of crops and cultures during the Bronze Age in Xinjiang (this map is edited by D.T. using ArcGIS 10.6 https://www.esri.com and Adobe Photoshop CC 14.0 https://www.adobe.com/products/photoshop.html, the base map is from World Terrain Base in ArcGIS Online https://www.arcgis.com/home/item.html?id=d476e726bd8c4c3aa5168d735f647dcc).

After 2000 BC, migrations on both a local and regional scale occurred across the Tianshan Mountains (Fig. 6). Adunqiaolu is located in the southern foothills of the Dzhungar (Alatao) Mountains, the northern spur of the Tianshan Range and one of the ridges of the IAMC. This region was suitable for pastoralism and increasing evidence points to herders as the main agents of cultural contact and crop dispersal in the Bronze Age^9,17^. Together with Adunqiaolu, the agro-pastoralist sites of Tasbas, Begash and Dali in eastern Kazakhstan are part of a Middle/Late Bronze Age cultural grouping that shares commonalities with the Federovo variant of the Andronovo Archaeological Complex (ca. 2000–900 BC)^27,31,58,71^.

Eastward movements intensified in the second millennium BC and the cultural impact of the Andronovo Archaeological Complex reached Central Asia and, further east, Xinjiang—specifically western and central Tianshan (e.g. Adunqiaolu, Husta, Kalasu, Saensayi), Pamir (Xiabandi), and the Altai Mountains (Xiakalangguer)^31,72–75^. Recent investigations at Baigetuobie suggest that the Andronovo influence may have extended as far as the eastern Tianshan Mountains^76^. The westward counterpart to these movements is represented by the so-called ‘painted pottery cultures’ in the Hexi Corridor—such as Siba and Xichengyi (ca. 2100–1500 BC)–which spread to eastern Xinjiang, as indicated by the remains at Tianshanbeilu and Nanwan^2,77,78^.

While climate fluctuations may have led to a diversification in cropping patterns, the dispersal of cereals was most likely driven by the local and regional migration of pastoralist communities across the Tianshan Mountains (Fig. 6). Cereal consumption by pastoralist groups may have begun as a supplementary subsistence strategy^21^. However, the change in dietary habits across time^22,64,65^ suggests that cereals were not exotica, but developed into a key element in local economic strategies. Crop dispersal was prompted by expediency, and quite possibly by necessity, which according to Liu and Jones^6^ was a primary agent of food globalization. These factors may have led to a shift in regional food production and consumption and to the adoption of new cereals as staple food.

The data sets presented and discussed in this study highlight a relative paucity of evidence for cereals, supporting the contention that House F1 and its neighbors were winter pastoral encampments. We hold that the upper reaches of the Boertala River Valley were seasonal pastures only, while crops were possibly cultivated in the lower reaches of the river. At the same time, we would also argue that the variety of grains in Adunqiaolu, including carbonized foxtail millet, broomcorn millet and naked barley in low density, was a consequence of the interconnectivity across the Tianshan Mountains during the second millennium BC. In particular, local and regional-scale migrations of pastoralists—the eastward expansion of the Andronovo Archaeological Complex and the westward movements of ‘the painted pottery cultures’ – facilitated crop dispersal in the Bronze Age.

## Methods

In this study, all methods comply with relevant institutional, national, and international guidelines and legislation.

### Archaeobotanical analyses

The plant material analyzed for this study was obtained during field excavations undertaken under a permit issued by the Chinese Academy of Social Sciences to the author D.C. All the samples were carbonized plant remains which do not constitute any biohazard or environmental threat. Carbonized plant remains do not fall under any specific regulations regarding laboratory analysis.

During the excavation of F1, 1 m × 1 m square grids were set up (Fig. 2). Two test trenches were set in the northeastern side of F1 to analyze the stratigraphy. 110 sediment samples (approximately 1492 L) were collected. All samples were floated at the excavation site, the protocol having been adapted from Pearsall^79^ and Zhao^80^. We filled a bucket with 10 L of water and sprinkled the soil sample evenly into the bucket, stirring gently with a plastic stick. The supernatant was poured into the second bucket through a 0.2 mm sampling sieve, and the process was repeated twice. Finally, the material collected in the sieve was transferred to the white cloth by spraying water, and it was then hung up and dried in the shade. The laboratory analysis was conducted at the Institute of Archaeology, Chinese Academy of Social Science, on the basis of Zhao^80^, with several references to Zhang^81^ and Liu et al.^82^. The samples were first screened in the laboratory with 2.0 mm, 1.0 mm and 0.5 mm sampling sieves, then observed and identified using a Cewei PXS-5T stereomicroscope, and photographed and measured using a Zeiss Stemi 2000C stereomicroscope. For botanical names, this paper employs the standard version recorded on the online Flora of China database (http://www.iplant.cn/foc/). The density of plant is the ratio of the number of plant seeds and the volume of sample (Table S2) and it illustrated by the software ArcGIS 10.6 (Fig. 5).

### Radiocarbon dating

Three samples of carbonized seeds selected for dating by AMS. Dates were measured at Beta Analytic laboratories in Miami (USA) and calibrated in OxCal 4.3, using IntCal13 calibration curve^83,84^.

### Carbon stable isotope

Three samples of carbonized barley were chosen for carbon stable isotope measurement. δ^13^C values were measured at the Beta Analytic laboratories in Miami (USA). In order to evaluate the water supply available to our barley during the growing period, the δ^13^C data were plugged into the formula by Farquhar et al.^85^:

$$ \Delta ^{{13}} {\text{C}}\left( \textperthousand \right) = \left( {\frac{{\delta ^{{13}} {\text{C}}_{{{\text{air}}}}  - \delta ^{{13}} {\text{C}}_{{{\text{plant}}}} }}{{1 + \delta ^{{13}} {\text{C}}_{{{\text{plant}}}} }}} \right) $$

δ^13^C_air_(δ^13^C of the atmosphere in the past) was established on the basis of Ferrio et al.^86^ (the dataset is available at http://web.udl.es/usuaris/x3845331/AIRCO2_LOESS.xls). δ^13^C_plant_ values were provided by the Beta Analytic laboratories in Miami (USA). The evaluation of the water supply during the growing period was made by referring to Wallace et al.^47^.

## Supplementary Information

Supplementary Information.
