# Supplementary material for: New evidence for supplementary crop production, foddering and fuel use by Bronze Age transhumant pastoralists in the Tianshan Mountains
Source: Sci Rep. 2021 Jul 2;11:13718. doi: 10.1038/s41598-021-93090-2 (PMC8253771; doi:10.1038/s41598-021-93090-2)
Supplement: Supplementary file 1 — Supplementary Information. [file 41598_2021_93090_MOESM1_ESM.docx]

# New evidence for supplementary crop production, foddering and fuel use by Bronze Age transhumant pastoralists in the Tianshan Mountains

Duo Tian^1,2,*^, Marcella Festa^2^, Dexin Cong^3^, Zhijun Zhao^2,3^, Peter Weiming Jia^4 5^, Alison Betts^4^

1 Institute of Middle Eastern Studies, Northwest University, Xi’an, Shaanxi 710127, P.R.China

2 School of Cultural Heritage, Northwest University, Xi’an, Shaanxi 710127, P.R.China

3 Institute of Archaeology, Chinese Academy of Social Sciences, Beijing 100010, P.R. China

4 Department of Archaeology and China Studies Centre, University of Sydney, NSW 2006, Australia

5 School of History and Culture, Henan University, Kaifeng 475001, China

* Corresponding author (Email: [tianduo@nwu.edu.cn](mailto:tianduo@nwu.edu.cn))

**Table S1.** The archaeobotanical material from Adunqiaolu F1

| Sampling grid | Sample volume(L) | Charcoal >2mm (g) | *S. italica* | *P. miliaceum* | *H.vulgare* (intact) | *H.vulgare* (fragmented) | *S. viridis* | *A. fatua* | *Carex* sp. | *Astragalus* sp. | *Chenopodium* | *Salsola* sp. | *Atriplex* sp. | *Rumex* sp. | *Galium* spp. | *Valeriana* sp. | Unknown |
| --- | --- | --- | --- | --- | --- | --- | --- | --- | --- | --- | --- | --- | --- | --- | --- | --- | --- |
| A07 | 8 | 14.75 |  |  |  |  |  |  |  |  |  |  |  |  |  |  |  |
| A08 | 8 | 1.23 |  |  |  |  |  |  |  |  |  |  |  |  |  |  |  |
| A09 | 16 | 2.31 |  |  |  |  |  |  |  | 7 |  |  |  |  |  |  |  |
| A10 | 8 | 0.08 |  |  |  |  |  |  |  |  |  |  |  |  |  |  |  |
| A11 | 8 | 0.02 |  |  |  |  |  |  |  |  |  |  |  |  |  |  |  |
| B02 | 8 | 0.71 |  |  |  |  |  |  |  |  |  |  |  |  |  |  |  |
| B03 | 8 | 0.75 |  |  |  | 1 |  |  |  | 4 |  |  |  |  |  |  |  |
| B04 | 8 | 0.86 |  |  |  | 2 |  |  |  |  |  |  |  |  |  | 1 |  |
| B05 | 8 | 2.16 |  |  |  |  |  |  |  | 2 |  |  |  |  |  |  |  |
| B07 | 8 | 3.95 |  |  |  |  |  |  |  | 3 | 1 |  | 1 |  |  |  |  |
| B08 | 8 | 7.86 |  |  |  |  |  |  |  |  |  |  |  |  |  |  |  |
| B09 | 16 | 1.79 |  |  |  |  |  |  |  | 9 |  |  |  |  |  |  |  |
| B10 | 8 | 0.34 |  |  |  |  |  |  |  |  |  |  |  |  |  |  |  |
| B11 | 8 | 0.14 |  |  |  |  |  |  |  |  |  | 1 |  |  |  |  |  |
| C02 | 8 | 0.61 |  |  | 1 |  |  |  |  |  |  |  |  |  |  |  |  |
| C03 | 8 | 3.16 |  |  |  |  |  |  |  | 1 |  |  |  |  |  |  |  |
| C04 | 8 | 1.90 |  |  | 1 |  |  |  |  | 1 |  |  |  |  |  |  |  |
| C05 | 8 | 2.18 |  |  |  |  |  | 1 |  | 1 |  |  |  |  |  |  |  |
| C09 | 16 | 0.21 |  |  |  |  |  |  | 2 | 11 |  |  |  |  |  |  |  |

Table S1 (Continued)

| Sampling grid | Sample volume(L) | Charcoal >2mm (g) | *S. italica* | *P. miliaceum* | *H.vulgare* (intact) | *H.vulgare* (fragmented) | *S. viridis* | *A. fatua* | *Carex* sp. | *Astragalus* sp. | *Chenopodium* | *Salsola* sp. | *Atriplex* sp. | *Rumex* sp. | *Galium* spp. | *Valeriana* sp. | Unknown |
| --- | --- | --- | --- | --- | --- | --- | --- | --- | --- | --- | --- | --- | --- | --- | --- | --- | --- |
| D02 | 8 | 1.24 |  |  |  |  |  |  |  | 1 |  |  |  |  |  |  |  |
| D03 | 8 | 2.13 |  |  |  |  |  |  |  | 2 |  |  |  |  |  |  |  |
| D04 | 8 | 2.08 |  |  |  |  |  |  |  | 2 | 1 |  |  |  |  |  |  |
| D05 | 8 | 2.50 |  |  |  |  |  |  |  |  |  |  |  |  |  |  |  |
| D13 | 8 | 1.03 |  |  |  |  |  | 1 |  | 5 | 1 |  |  |  |  |  |  |
| E02 | 8 | 1.63 |  |  |  |  |  |  |  | 1 |  |  |  |  |  |  |  |
| E03 | 8 | 0.06 |  |  |  |  |  |  |  |  |  |  |  |  |  |  |  |
| E04 | 8 | 0.49 |  |  |  |  |  |  |  |  |  |  |  |  |  |  |  |
| E07 | 8 | 0.66 |  |  |  |  |  | 1 |  |  |  |  |  |  |  |  |  |
| E08 | 8 | 0.92 |  |  |  |  |  |  |  | 2 |  |  |  |  |  |  |  |
| E09 | 8 | 0.20 |  |  |  |  |  |  |  |  |  |  |  |  |  |  |  |
| E10 | 8 | 1.06 |  |  |  |  |  |  |  | 1 |  |  |  |  |  |  |  |
| E11 | 8 | 0.09 |  |  |  |  |  |  |  |  |  |  |  |  |  |  |  |
| E12 | 8 | 0.07 |  |  |  |  |  |  |  |  |  |  |  |  |  |  |  |
| E13 | 8 | 0.03 |  |  |  |  |  |  |  |  |  |  |  |  |  |  |  |
| E14 | 16 | 0.01 |  |  |  |  |  |  |  |  |  |  |  |  |  |  |  |
| G02 | 8 | 1.06 |  |  | 1 |  |  |  |  | 1 | 2 |  |  | 1 |  |  |  |
| H04 | 8 | 0.55 |  |  |  |  |  |  |  | 2 | 1 |  |  |  |  |  |  |
| H07 | 8 | 2.61 |  |  |  |  |  |  |  |  | 2 |  |  |  |  |  |  |

Table S1 (Continued)

| Sampling grid | Sample volume(L) | Charcoal >2mm (g) | *S. italica* | *P. miliaceum* | *H.vulgare* (intact) | *H.vulgare* (fragmented) | *S. viridis* | *A. fatua* | *Carex* sp. | *Astragalus* sp. | *Chenopodium* | *Salsola* sp. | *Atriplex* sp. | *Rumex* sp. | *Galium* spp. | *Valeriana* sp. | Unknown |
| --- | --- | --- | --- | --- | --- | --- | --- | --- | --- | --- | --- | --- | --- | --- | --- | --- | --- |
| H08 | 8 | 0.20 |  |  |  |  |  |  |  |  |  |  |  |  |  |  |  |
| H10 | 8 | 0.11 |  |  |  |  |  |  |  | 1 |  |  |  |  |  |  |  |
| I01 | 8 | 0.78 |  |  |  | 1 |  |  |  |  |  |  |  |  |  |  |  |
| I02 | 8 | 0.84 |  |  |  |  |  |  |  | 2 |  |  |  |  |  |  |  |
| I05 | 8 | 0.23 |  |  |  |  |  |  |  |  |  |  |  |  |  |  |  |
| I06 | 8 | 0.02 |  |  |  |  |  |  |  | 2 |  |  |  |  |  |  |  |
| I07 | 8 | 0.36 |  |  |  |  |  |  |  |  |  |  |  |  |  |  |  |
| I08 | 8 | 0.42 |  |  | 2 | 1 |  |  |  | 2 | 2 |  |  |  |  |  |  |
| I10 | 8 | 0.48 |  |  |  |  |  |  |  | 2 |  |  |  |  |  |  |  |
| J01 | 8 | 0.90 |  |  | 1 |  |  |  |  | 4 |  |  |  |  |  |  |  |
| J02 | 24 | 0.53 |  |  |  |  |  |  |  | 2 |  |  |  |  |  |  |  |
| J03 | 16 | 0.94 |  |  |  |  |  |  |  |  |  |  |  |  |  |  |  |
| J04 | 8 | 0.82 |  | 1 |  |  |  |  |  | 1 |  |  |  |  |  |  |  |
| J05 | 8 | 0.02 |  |  |  |  |  |  |  |  |  |  |  |  |  |  |  |
| J06 | 8 | 0.00 |  |  |  |  |  |  |  |  |  |  |  |  |  |  |  |
| J08 | 8 | 0.19 |  |  |  |  |  |  |  | 2 |  |  |  |  |  |  |  |
| K04 | 8 | 1.02 |  |  |  |  |  |  |  | 2 |  |  |  |  |  |  |  |
| K05 | 16 | 0.03 |  |  |  |  |  |  |  |  |  |  |  |  |  |  |  |
| K06 | 32 | 0.44 | 2 |  |  |  |  |  |  | 14 | 11 |  |  |  |  |  |  |

Table S1 (Continued)

| Sampling grid | Sample volume(L) | Charcoal >2mm (g) | *S. italica* | *P. miliaceum* | *H.vulgare* (intact) | *H.vulgare* (fragmented) | *S. viridis* | *A. fatua* | *Carex* sp. | *Astragalus* sp. | *Chenopodium* | *Salsola* sp. | *Atriplex* sp. | *Rumex* sp. | *Galium* spp. | *Valeriana* sp. | Unknown |
| --- | --- | --- | --- | --- | --- | --- | --- | --- | --- | --- | --- | --- | --- | --- | --- | --- | --- |
| K07 | 24 | 8.34 |  | 1 |  |  |  |  |  | 3 |  |  |  |  |  |  |  |
| K12 | 16 | 0.18 |  |  |  |  |  |  |  | 2 | 2 |  |  |  |  |  |  |
| L04 | 8 | 0.26 |  |  |  |  |  |  |  |  |  |  |  |  |  |  |  |
| L05 | 8 | 0.10 |  |  |  |  |  |  |  | 1 |  |  |  |  |  |  |  |
| L06 | 8 | 0.04 |  |  |  |  |  |  |  |  |  |  |  |  |  |  |  |
| L11 | 32 | 7.38 | 1 |  |  |  |  |  |  | 2 | 8 |  |  |  |  |  |  |
| L12 | 24 | 2.34 |  |  |  |  |  |  |  | 5 | 1 |  |  |  |  |  |  |
| L13 | 16 | 2.22 |  |  |  |  |  |  |  | 2 | 4 |  |  |  |  |  |  |
| L14 | 24 | 3.38 |  |  |  |  |  |  |  | 3 | 4 |  |  |  |  |  | 1 |
| L15 | 16 | 2.80 |  |  |  |  |  |  |  | 2 |  |  |  |  |  |  |  |
| M01 | 16 | 1.52 |  |  |  |  |  |  |  | 3 |  |  |  |  |  |  | 1 |
| M04 | 8 | 3.05 | 1 |  |  | 1 |  |  |  | 9 | 2 |  |  |  |  |  |  |
| M06 | 8 | 0.08 |  |  |  |  |  |  |  |  |  |  |  |  |  |  |  |
| M07 | 16 | 0.07 |  |  |  |  |  |  |  |  |  |  |  |  |  |  |  |
| M11 | 32 | 2.42 | 1 |  |  |  |  |  |  | 20 | 4 | 1 |  |  |  |  |  |
| M12 | 24 | 7.16 |  |  |  |  |  |  |  | 5 | 31 |  |  |  |  |  |  |
| M13 | 40 | 12.69 |  |  |  | 1 |  |  |  | 14 | 12 |  | 1 |  |  |  |  |
| M14 | 24 | 3.94 |  |  |  |  |  |  |  | 15 | 37 |  |  |  |  |  |  |
| M15 | 32 | 5.15 |  |  |  |  |  |  |  | 3 |  |  |  |  |  |  |  |

Table S1 (Continued)

| Sampling grid | Sample volume(L) | Charcoal >2mm (g) | *S. italica* | *P. miliaceum* | *H.vulgare* (intact) | *H.vulgare* (fragmented) | *S. viridis* | *A. fatua* | *Carex* sp. | *Astragalus* sp. | *Chenopodium* | *Salsola* sp. | *Atriplex* sp. | *Rumex* sp. | *Galium* spp. | *Valeriana* sp. | Unknown |
| --- | --- | --- | --- | --- | --- | --- | --- | --- | --- | --- | --- | --- | --- | --- | --- | --- | --- |
| N03 | 10 | 1.00 |  |  | 1 |  |  |  |  |  |  |  |  |  |  |  |  |
| N06 | 16 | 0.00 |  |  |  |  |  |  |  |  |  |  | 1 |  |  |  |  |
| N11 | 24 | 6.23 | 1 |  |  |  |  |  |  | 15 | 10 |  |  |  |  |  | 2 |
| N12 | 32 | 5.23 |  |  |  |  |  |  |  | 16 | 14 |  |  |  |  |  |  |
| N13 | 32 | 8.12 |  |  |  |  |  |  |  | 18 | 16 |  |  |  |  |  | 1 |
| N14 | 24 | 2.65 |  | 1 |  | 1 |  |  |  | 28 | 50 |  |  |  |  |  |  |
| N15 | 24 | 4.62 |  | 1 |  | 1 |  |  | 1 | 10 | 11 |  |  |  |  |  |  |
| O01 | 16 | 1.50 | 1 |  |  |  | 1 |  |  | 2 | 2 |  | 1 |  |  |  |  |
| O02 | 8 | 1.66 |  | 1 | 3 |  |  |  |  | 1 | 1 |  |  |  |  |  |  |
| O03 | 16 | 6.52 | 3 |  | 2 |  |  |  |  | 5 | 32 |  |  |  |  |  |  |
| O04 | 8 | 8.18 | 1 | 2 | 1 |  |  |  |  | 2 | 3 |  |  |  |  |  |  |
| O05 | 8 | 5.52 |  |  |  | 1 |  |  |  | 2 | 1 |  |  |  |  |  |  |
| O06 | 16 | 4.58 |  |  |  | 1 |  |  |  | 1 |  |  |  |  |  |  |  |
| O08 | 10 | 26.52 |  | 1 |  |  |  |  |  | 10 | 67 |  |  |  |  |  |  |
| O11 | 16 | 1.79 |  |  |  |  |  |  |  | 8 | 9 |  |  |  |  |  |  |
| O12 | 24 | 4.15 |  |  |  | 1 |  |  |  | 13 | 10 |  |  |  |  |  |  |
| O13 | 32 | 24.12 |  | 1 | 1 | 1 |  |  |  | 22 | 15 |  |  |  |  |  | 1 |
| O14 | 24 | 3.59 |  | 2 |  |  |  | 1 | 2 | 29 | 58 |  |  |  |  |  |  |
| O15 | 24 | 3.92 |  |  |  | 1 | 1 |  |  | 11 | 7 |  |  |  |  |  |  |

Table S1 (Continued)

| Sampling grid | Sample volume(L) | Charcoal >2mm (g) | *S. italica* | *P. miliaceum* | *H.vulgare* (intact) | *H.vulgare* (fragmented) | *S. viridis* | *A. fatua* | *Carex* sp. | *Astragalus* sp. | *Chenopodium* | *Salsola* sp. | *Atriplex* sp. | *Rumex* sp. | *Galium* spp. | *Valeriana* sp. | Unknown |
| --- | --- | --- | --- | --- | --- | --- | --- | --- | --- | --- | --- | --- | --- | --- | --- | --- | --- |
| P01 | 24 | 1.12 | 1 |  |  |  |  |  |  | 3 | 1 |  | 1 |  |  |  |  |
| P02 | 8 | 1.71 |  |  |  |  |  |  |  | 1 |  |  |  |  |  |  |  |
| P03 | 16 | 9.39 | 3 | 1 | 1 |  |  |  |  | 3 | 9 |  |  |  |  |  |  |
| P04 | 24 | 12.78 | 1 |  |  | 1 |  |  |  | 5 |  |  |  |  |  |  |  |
| P05 | 32 | 3.96 |  | 1 | 1 | 3 |  |  |  | 7 | 1 |  | 2 |  | 1 |  |  |
| P06 | 16 | 10.59 |  | 1 |  |  |  |  |  | 3 | 3 |  |  |  |  |  |  |
| P07 | 8 | 40.56 |  |  |  |  |  |  |  | 4 | 56 |  |  |  |  |  |  |
| P11 | 8 | 1.40 |  |  |  |  |  |  |  | 10 |  |  |  |  |  |  |  |
| P12 | 8 | 0.60 |  |  |  |  |  |  |  | 2 | 1 |  |  |  |  |  |  |
| P13 | 4 | 1.39 |  |  |  |  |  |  |  | 1 | 7 |  |  |  |  |  |  |
| P14 | 4 | 0.30 |  |  |  |  |  |  |  | 8 | 12 |  |  |  |  |  |  |
| Q05 | 8 | 1.99 | 1 | 1 |  |  |  |  |  | 5 | 18 |  | 2 |  |  |  |  |
| Q06 | 32 | 0.42 |  | 1 | 2 |  |  |  |  | 21 | 17 |  | 1 |  |  |  |  |
| Q07 | 8 | 8.52 |  |  | 1 |  |  |  |  | 18 | 44 |  |  |  |  |  |  |
| R14 | 8 | 0.09 |  |  |  |  |  | 1 |  | 2 |  |  |  |  | 1 |  |  |
| Total | 1492 | 349.61 | 17 | 16 | 19 | 19 | 2 | 5 | 5 | 468 | 601 | 2 | 10 | 1 | 2 | 1 | 6 |

**Table S2.** The density of charcoal, barley, millet, *Astragalus* and *Chenopodium* in F1

| Sampling  grid | Sample  volume(L) | Charcoal | | Barley | | Foxtail millet | | Broomcorn millet | | *Astragalus* | | *Chenopodium* | |
| --- | --- | --- | --- | --- | --- | --- | --- | --- | --- | --- | --- | --- | --- |
|  |  | Quantity | Density | Quantity | Density | Quantity | Density | Quantity | Density | Quantity | Density | Quantity | Density |
| A07 | 8 | 14.75 | 1.84 |  | 0.00 |  | 0.00 |  | 0.00 |  | 0.00 |  | 0.00 |
| A08 | 8 | 1.23 | 0.15 |  | 0.00 |  | 0.00 |  | 0.00 |  | 0.00 |  | 0.00 |
| A09 | 16 | 2.31 | 0.14 |  | 0.00 |  | 0.00 |  | 0.00 | 7 | 0.44 |  | 0.00 |
| A10 | 8 | 0.08 | 0.01 |  | 0.00 |  | 0.00 |  | 0.00 |  | 0.00 |  | 0.00 |
| A11 | 8 | 0.02 | 0.00 |  | 0.00 |  | 0.00 |  | 0.00 |  | 0.00 |  | 0.00 |
| B02 | 8 | 0.71 | 0.09 |  | 0.00 |  | 0.00 |  | 0.00 |  | 0.00 |  | 0.00 |
| B03 | 8 | 0.75 | 0.09 | 1 | 0.13 |  | 0.00 |  | 0.00 | 4 | 0.50 |  | 0.00 |
| B04 | 8 | 0.86 | 0.11 | 2 | 0.25 |  | 0.00 |  | 0.00 |  | 0.00 |  | 0.00 |
| B05 | 8 | 2.16 | 0.27 |  | 0.00 |  | 0.00 |  | 0.00 | 2 | 0.25 |  | 0.00 |
| B07 | 8 | 3.95 | 0.49 |  | 0.00 |  | 0.00 |  | 0.00 | 3 | 0.38 | 1 | 0.13 |
| B08 | 8 | 7.86 | 0.98 |  | 0.00 |  | 0.00 |  | 0.00 |  | 0.00 |  | 0.00 |
| B09 | 16 | 1.79 | 0.11 |  | 0.00 |  | 0.00 |  | 0.00 | 9 | 0.56 |  | 0.00 |
| B10 | 8 | 0.34 | 0.04 |  | 0.00 |  | 0.00 |  | 0.00 |  | 0.00 |  | 0.00 |
| B11 | 8 | 0.14 | 0.02 |  | 0.00 |  | 0.00 |  | 0.00 |  | 0.00 |  | 0.00 |
| C02 | 8 | 0.61 | 0.08 | 1 | 0.13 |  | 0.00 |  | 0.00 |  | 0.00 |  | 0.00 |
| C03 | 8 | 3.16 | 0.40 |  | 0.00 |  | 0.00 |  | 0.00 | 1 | 0.13 |  | 0.00 |
| C04 | 8 | 1.90 | 0.24 | 1 | 0.13 |  | 0.00 |  | 0.00 | 1 | 0.13 |  | 0.00 |
| C05 | 8 | 2.18 | 0.27 |  | 0.00 |  | 0.00 |  | 0.00 | 1 | 0.13 |  | 0.00 |
| C09 | 16 | 0.21 | 0.01 |  | 0.00 |  | 0.00 |  | 0.00 | 11 | 0.69 |  | 0.00 |
|  |  |  |  |  |  |  |  |  |  |  |  |  |  |

Table S2 (Continued)

| Sampling  grid | Sample  volume(L) | Charcoal | | Barley | | Foxtail millet | | Broomcorn millet | | *Astragalus* | | *Chenopodium* | |
| --- | --- | --- | --- | --- | --- | --- | --- | --- | --- | --- | --- | --- | --- |
|  |  | Quantity | Density | Quantity | Density | Quantity | Density | Quantity | Density | Quantity | Density | Quantity | Density |
| D02 | 8 | 1.24 | 0.16 |  | 0.00 |  | 0.00 |  | 0.00 | 1 | 0.13 |  | 0.00 |
| D03 | 8 | 2.13 | 0.27 |  | 0.00 |  | 0.00 |  | 0.00 | 2 | 0.25 |  | 0.00 |
| D04 | 8 | 2.08 | 0.26 |  | 0.00 |  | 0.00 |  | 0.00 | 2 | 0.25 | 1 | 0.13 |
| D05 | 8 | 2.50 | 0.31 |  | 0.00 |  | 0.00 |  | 0.00 |  | 0.00 |  | 0.00 |
| D13 | 8 | 1.03 | 0.13 |  | 0.00 |  | 0.00 |  | 0.00 | 5 | 0.63 | 1 | 0.13 |
| E02 | 8 | 1.63 | 0.20 |  | 0.00 |  | 0.00 |  | 0.00 | 1 | 0.13 |  | 0.00 |
| E03 | 8 | 0.06 | 0.01 |  | 0.00 |  | 0.00 |  | 0.00 |  | 0.00 |  | 0.00 |
| E04 | 8 | 0.49 | 0.06 |  | 0.00 |  | 0.00 |  | 0.00 |  | 0.00 |  | 0.00 |
| E07 | 8 | 0.66 | 0.08 |  | 0.00 |  | 0.00 |  | 0.00 |  | 0.00 |  | 0.00 |
| E08 | 8 | 0.92 | 0.12 |  | 0.00 |  | 0.00 |  | 0.00 | 2 | 0.25 |  | 0.00 |
| E09 | 8 | 0.20 | 0.02 |  | 0.00 |  | 0.00 |  | 0.00 |  | 0.00 |  | 0.00 |
| E10 | 8 | 1.06 | 0.13 |  | 0.00 |  | 0.00 |  | 0.00 | 1 | 0.13 |  | 0.00 |
| E11 | 8 | 0.09 | 0.01 |  | 0.00 |  | 0.00 |  | 0.00 |  | 0.00 |  | 0.00 |
| E12 | 8 | 0.07 | 0.01 |  | 0.00 |  | 0.00 |  | 0.00 |  | 0.00 |  | 0.00 |
| E13 | 8 | 0.03 | 0.00 |  | 0.00 |  | 0.00 |  | 0.00 |  | 0.00 |  | 0.00 |
| E14 | 16 | 0.01 | 0.00 | 1 | 0.06 |  | 0.00 |  | 0.00 | 3 | 0.19 |  | 0.00 |
| G02 | 8 | 1.06 | 0.13 | 1 | 0.13 |  | 0.00 |  | 0.00 | 1 | 0.13 | 2 | 0.25 |
| H04 | 8 | 0.55 | 0.07 |  | 0.00 |  | 0.00 |  | 0.00 | 2 | 0.25 | 1 | 0.13 |
| H07 | 8 | 2.61 | 0.33 |  | 0.00 |  | 0.00 |  | 0.00 |  | 0.00 | 2 | 0.25 |
|  |  |  |  |  |  |  |  |  |  |  |  |  |  |

Table S2 (Continued)

| Sampling  grid | Sample  volume(L) | Charcoal | | Barley | | Foxtail millet | | Broomcorn millet | | *Astragalus* | | *Chenopodium* | |
| --- | --- | --- | --- | --- | --- | --- | --- | --- | --- | --- | --- | --- | --- |
|  |  | Quantity | Density | Quantity | Density | Quantity | Density | Quantity | Density | Quantity | Density | Quantity | Density |
| H08 | 8 | 0.20 | 0.03 |  | 0.00 |  | 0.00 |  | 0.00 |  | 0.00 |  | 0.00 |
| H10 | 8 | 0.11 | 0.01 |  | 0.00 |  | 0.00 |  | 0.00 | 1 | 0.13 |  | 0.00 |
| I01 | 8 | 0.78 | 0.10 | 1 | 0.13 |  | 0.00 |  | 0.00 |  | 0.00 |  | 0.00 |
| I02 | 8 | 0.84 | 0.11 |  | 0.00 |  | 0.00 |  | 0.00 | 2 | 0.25 |  | 0.00 |
| I05 | 8 | 0.23 | 0.03 |  | 0.00 |  | 0.00 |  | 0.00 |  | 0.00 |  | 0.00 |
| I06 | 8 | 0.02 | 0.00 |  | 0.00 |  | 0.00 |  | 0.00 | 2 | 0.25 |  | 0.00 |
| I07 | 8 | 0.36 | 0.04 |  | 0.00 |  | 0.00 |  | 0.00 |  | 0.00 |  | 0.00 |
| I08 | 8 | 0.42 | 0.05 | 3 | 0.38 |  | 0.00 |  | 0.00 | 2 | 0.25 | 2 | 0.25 |
| I10 | 8 | 0.48 | 0.06 |  | 0.00 |  | 0.00 |  | 0.00 | 2 | 0.25 |  | 0.00 |
| J01 | 8 | 0.90 | 0.11 | 1 | 0.13 |  | 0.00 |  | 0.00 | 4 | 0.50 |  | 0.00 |
| J02 | 24 | 0.53 | 0.02 |  | 0.00 |  | 0.00 |  | 0.00 | 2 | 0.08 |  | 0.00 |
| J03 | 16 | 0.94 | 0.06 |  | 0.00 |  | 0.00 |  | 0.00 |  | 0.00 |  | 0.00 |
| J04 | 8 | 0.82 | 0.10 |  | 0.00 |  | 0.00 | 1 | 0.13 | 1 | 0.13 |  | 0.00 |
| J05 | 8 | 0.02 | 0.00 |  | 0.00 |  | 0.00 |  | 0.00 |  | 0.00 |  | 0.00 |
| J06 | 8 | 0.00 | 0.00 |  | 0.00 |  | 0.00 |  | 0.00 |  | 0.00 |  | 0.00 |
| J08 | 8 | 0.19 | 0.02 |  | 0.00 |  | 0.00 |  | 0.00 | 2 | 0.25 |  | 0.00 |
| K04 | 8 | 1.02 | 0.13 |  | 0.00 |  | 0.00 |  | 0.00 | 2 | 0.25 |  | 0.00 |
| K05 | 16 | 0.03 | 0.00 |  | 0.00 |  | 0.00 |  | 0.00 |  | 0.00 |  | 0.00 |
| K06 | 32 | 0.44 | 0.01 |  | 0.00 | 2 | 0.06 |  | 0.00 | 14 | 0.44 | 11 | 0.34 |
|  |  |  |  |  |  |  |  |  |  |  |  |  |  |

Table S2 (Continued)

| Sampling  grid | Sample  volume(L) | Charcoal | | Barley | | Foxtail millet | | Broomcorn millet | | *Astragalus* | | *Chenopodium* | |
| --- | --- | --- | --- | --- | --- | --- | --- | --- | --- | --- | --- | --- | --- |
|  |  | Quantity | Density | Quantity | Density | Quantity | Density | Quantity | Density | Quantity | Density | Quantity | Density |
| K07 | 24 | 8.34 | 0.35 |  | 0.00 |  | 0.00 | 1 | 0.04 | 3 | 0.13 |  | 0.00 |
| K12 | 16 | 0.18 | 0.01 |  | 0.00 |  | 0.00 |  | 0.00 | 2 | 0.13 | 2 | 0.13 |
| L04 | 8 | 0.26 | 0.03 |  | 0.00 |  | 0.00 |  | 0.00 |  | 0.00 |  | 0.00 |
| L05 | 8 | 0.10 | 0.01 |  | 0.00 |  | 0.00 |  | 0.00 | 1 | 0.13 |  | 0.00 |
| L06 | 8 | 0.04 | 0.00 |  | 0.00 |  | 0.00 |  | 0.00 |  | 0.00 |  | 0.00 |
| L11 | 32 | 7.38 | 0.23 |  | 0.00 | 1 | 0.03 |  | 0.00 | 2 | 0.06 | 8 | 0.25 |
| L12 | 24 | 2.34 | 0.10 |  | 0.00 |  | 0.00 |  | 0.00 | 5 | 0.21 | 1 | 0.04 |
| L13 | 16 | 2.22 | 0.14 |  | 0.00 |  | 0.00 |  | 0.00 | 2 | 0.13 | 4 | 0.25 |
| L14 | 24 | 3.38 | 0.14 |  | 0.00 |  | 0.00 |  | 0.00 | 3 | 0.13 | 4 | 0.17 |
| L15 | 16 | 2.80 | 0.18 |  | 0.00 |  | 0.00 |  | 0.00 | 2 | 0.13 |  | 0.00 |
| M01 | 16 | 1.52 | 0.10 |  | 0.00 |  | 0.00 |  | 0.00 | 3 | 0.19 |  | 0.00 |
| M04 | 8 | 3.05 | 0.38 | 1 | 0.13 | 1 | 0.13 |  | 0.00 | 9 | 1.13 | 2 | 0.25 |
| M06 | 8 | 0.08 | 0.01 |  | 0.00 |  | 0.00 |  | 0.00 |  | 0.00 |  | 0.00 |
| M07 | 16 | 0.07 | 0.00 |  | 0.00 |  | 0.00 |  | 0.00 |  | 0.00 |  | 0.00 |
| M11 | 32 | 2.42 | 0.08 |  | 0.00 | 1 | 0.03 |  | 0.00 | 20 | 0.63 | 4 | 0.13 |
| M12 | 24 | 7.16 | 0.30 |  | 0.00 |  | 0.00 |  | 0.00 | 5 | 0.21 | 31 | 1.29 |
| M13 | 40 | 12.69 | 0.32 | 1 | 0.03 |  | 0.00 |  | 0.00 | 14 | 0.35 | 12 | 0.30 |
| M14 | 24 | 3.94 | 0.16 |  | 0.00 |  | 0.00 |  | 0.00 | 15 | 0.63 | 37 | 1.54 |
| M15 | 32 | 5.15 | 0.16 |  | 0.00 |  | 0.00 |  | 0.00 | 3 | 0.09 |  | 0.00 |
|  |  |  |  |  |  |  |  |  |  |  |  |  |  |

Table S2 (Continued)

| Sampling  grid | Sample  volume(L) | Charcoal | | Barley | | Foxtail millet | | Broomcorn millet | | *Astragalus* | | *Chenopodium* | |
| --- | --- | --- | --- | --- | --- | --- | --- | --- | --- | --- | --- | --- | --- |
|  |  | Quantity | Density | Quantity | Density | Quantity | Density | Quantity | Density | Quantity | Density | Quantity | Density |
| N03 | 10 | 1.00 | 0.10 | 1 | 0.10 |  | 0.00 |  | 0.00 |  | 0.00 |  | 0.00 |
| N06 | 16 | 0.00 | 0.00 |  | 0.00 |  | 0.00 |  | 0.00 |  | 0.00 |  | 0.00 |
| N11 | 24 | 6.23 | 0.26 |  | 0.00 | 1 | 0.04 |  | 0.00 | 15 | 0.63 | 10 | 0.42 |
| N12 | 32 | 5.23 | 0.16 |  | 0.00 |  | 0.00 |  | 0.00 | 16 | 0.50 | 14 | 0.44 |
| N13 | 32 | 8.12 | 0.25 |  | 0.00 |  | 0.00 |  | 0.00 | 18 | 0.56 | 16 | 0.50 |
| N14 | 24 | 2.65 | 0.11 | 1 | 0.04 |  | 0.00 | 1 | 0.04 | 28 | 1.17 | 50 | 2.08 |
| N15 | 24 | 4.62 | 0.19 | 1 | 0.04 |  | 0.00 | 1 | 0.04 | 10 | 0.42 | 11 | 0.46 |
| O01 | 16 | 1.50 | 0.09 |  | 0.00 | 1 | 0.06 |  | 0.00 | 2 | 0.13 | 2 | 0.13 |
| O02 | 8 | 1.66 | 0.21 | 3 | 0.38 |  | 0.00 | 1 | 0.13 | 1 | 0.13 | 1 | 0.13 |
| O03 | 16 | 6.52 | 0.41 | 2 | 0.13 | 3 | 0.19 |  | 0.00 | 5 | 0.31 | 32 | 2.00 |
| O04 | 8 | 8.18 | 1.02 | 1 | 0.13 | 1 | 0.13 | 2 | 0.25 | 2 | 0.25 | 3 | 0.38 |
| O05 | 8 | 5.52 | 0.69 | 1 | 0.13 |  | 0.00 |  | 0.00 | 2 | 0.25 | 1 | 0.13 |
| O06 | 16 | 4.58 | 0.29 | 1 | 0.06 |  | 0.00 |  | 0.00 | 1 | 0.06 |  | 0.00 |
| O08 | 10 | 26.52 | 2.65 |  | 0.00 |  | 0.00 | 1 | 0.10 | 10 | 1.00 | 67 | 6.70 |
| O11 | 16 | 1.79 | 0.11 |  | 0.00 |  | 0.00 |  | 0.00 | 8 | 0.50 | 9 | 0.56 |
| O12 | 24 | 4.15 | 0.17 | 1 | 0.04 |  | 0.00 |  | 0.00 | 13 | 0.54 | 10 | 0.42 |
| O13 | 32 | 24.12 | 0.75 | 2 | 0.06 |  | 0.00 | 1 | 0.03 | 22 | 0.69 | 15 | 0.47 |
| O14 | 24 | 3.59 | 0.15 |  | 0.00 |  | 0.00 | 2 | 0.08 | 29 | 1.21 | 58 | 2.42 |
| O15 | 24 | 3.92 | 0.16 | 1 | 0.04 |  | 0.00 |  | 0.00 | 11 | 0.46 | 7 | 0.29 |
|  |  |  |  |  |  |  |  |  |  |  |  |  |  |

Table S2 (Continued)

| Sampling  grid | Sample  volume(L) | Charcoal | | Barley | | Foxtail millet | | Broomcorn millet | | *Astragalus* | | *Chenopodium* | |
| --- | --- | --- | --- | --- | --- | --- | --- | --- | --- | --- | --- | --- | --- |
|  |  | Quantity | Density | Quantity | Density | Quantity | Density | Quantity | Density | Quantity | Density | Quantity | Density |
| P01 | 24 | 1.12 | 0.05 |  | 0.00 | 1 | 0.04 |  | 0.00 | 3 | 0.13 | 1 | 0.04 |
| P02 | 8 | 1.71 | 0.21 |  | 0.00 |  | 0.00 |  | 0.00 | 1 | 0.13 |  | 0.00 |
| P03 | 16 | 9.39 | 0.59 |  | 0.06 | 3 | 0.19 | 1 | 0.06 | 3 | 0.19 | 9 | 0.56 |
| P04 | 24 | 12.78 | 0.53 | 1 | 0.04 | 1 | 0.04 |  | 0.00 | 5 | 0.21 |  | 0.00 |
| P05 | 32 | 3.96 | 0.12 | 4 | 0.13 |  | 0.00 | 1 | 0.03 | 7 | 0.22 | 1 | 0.03 |
| P06 | 16 | 10.59 | 0.66 |  | 0.00 |  | 0.00 | 1 | 0.06 | 3 | 0.19 | 3 | 0.19 |
| P07 | 8 | 40.56 | 5.07 |  | 0.00 |  | 0.00 |  | 0.00 | 4 | 0.50 | 56 | 7.00 |
| P11 | 8 | 1.40 | 0.18 |  | 0.00 |  | 0.00 |  | 0.00 | 10 | 1.25 |  | 0.00 |
| P12 | 8 | 0.60 | 0.08 |  | 0.00 |  | 0.00 |  | 0.00 | 2 | 0.25 | 1 | 0.13 |
| P13 | 4 | 1.39 | 0.35 |  | 0.00 |  | 0.00 |  | 0.00 | 1 | 0.25 | 7 | 1.75 |
| P14 | 4 | 0.30 | 0.08 |  | 0.00 |  | 0.00 |  | 0.00 | 8 | 2.00 | 12 | 3.00 |
| Q05 | 8 | 1.99 | 0.25 |  | 0.00 | 1 | 0.13 | 1 | 0.13 | 5 | 0.63 | 18 | 2.25 |
| Q06 | 32 | 0.42 | 0.01 | 2 | 0.06 |  | 0.00 | 1 | 0.03 | 21 | 0.66 | 17 | 0.53 |
| Q07 | 8 | 8.52 | 1.07 | 1 | 0.13 |  | 0.00 |  | 0.00 | 18 | 2.25 | 44 | 5.50 |
| R14 | 8 | 0.09 | 0.01 |  | 0.00 |  | 0.00 |  | 0.00 | 2 | 0.25 |  | 0.00 |
|  |  |  |  |  |  |  |  |  |  |  |  |  |  |

**Table S3. Measurements of barley grains**

| Sampling grid | Length (mm) | Width (mm) | Thickness (mm) |
| --- | --- | --- | --- |
| I08 | 5.39 | 3.25 | 2.62 |
| I08 | 4.15 | 2.85 | 2.06 |
| J01 | 4.21 | 2.25 | 1.95 |
| O02 | 4.7 | 3.11 | 1.91 |
| O02 | 3.68 | 2.73 | 1.76 |
| O02 | 2.94 | 2.1 | 1.33 |
| O03 | 4.79 | 3.3 | 2.35 |
| O03 | 4.7 | 3.03 | 2.1 |
| Q06 | 4.52 | 2.81 | 2.1 |
| Q06 | 3.68 | 2.67 | 1.81 |
| Q07 | 3.93 | 3.03 | 1.77 |
| Average | 4.26 | 2.81 | 1.99 |
| SDEV | 0.65 | 0.37 | 0.32 |
|  |  |  |  |

**Table S4. Measurements of broomcorn millet grains**

| Sampling grid | Length (mm) | Width (mm) |
| --- | --- | --- |
| J4 | 1.95 | 1.89 |
| K7 | 2.08 | 2.02 |
| N14 | 2.07 | 2.01 |
| N15 | 1.77 | 1.68 |
| O2 | 2.08 | 1.93 |
| O4 | 1.83 | 1.83 |
| O4 | 1.45 | 1.15 |
| O8 | 2.12 | 1.75 |
| O14 | 1.94 | 1.66 |
| P6 | 2.19 | 1.9 |
| Q5 | 1.89 | 1.77 |
| Average | 1.94 | 1.78 |
| SDEV | 0.21 | 0.24 |
|  |  |  |

**Table S5. Measurements of foxtail millet grains**

| Sampling grid | Length (mm) | Width (mm) |
| --- | --- | --- |
| K6 | 1.49 | 1.26 |
| L11 | 1.3 | 1.21 |
| M4 | 1.53 | 1.44 |
| N11 | 1.24 | 0.99 |
| O1 | 1.56 | 1.39 |
| O3 | 1.72 | 1.34 |
| O3 | 1.72 | 1.53 |
| O4 | 1.47 | 1.35 |
| P1 | 1.49 | 1.31 |
| P4 | 1.3 | 1.26 |
| Q5 | 1.51 | 1.3 |
| Average | 1.48 | 1.31 |
| SDEV | 0.16 | 0.14 |
|  |  |  |
